# A Systematic Review on Viruses in Mass-Reared Edible Insect Species

**DOI:** 10.3390/v13112280

**Published:** 2021-11-15

**Authors:** Michela Bertola, Franco Mutinelli

**Affiliations:** 1 Laboratory of Parasitology Micology and Sanitary Enthomology, Istituto Zooprofilattico Sperimentale delle Venezie, Viale dell’Università 10, 35020 Legnaro, PD, Italy; 2 National Rereference Laboratory for Honey Bee Health, Istituto Zooprofilattico Sperimentale delle Venezie, Viale dell’Università 10, 35020 Legnaro, PD, Italy; fmutinelli@izsvenezie.it

**Keywords:** edible insects, mass rearing, production system, controlled environment, virus, regulation

## Abstract

Edible insects are expected to become an important nutrient source for animals and humans in the Western world in the near future. Only a few studies on viruses in edible insects with potential for industrial rearing have been published and concern only some edible insect species. Viral pathogens that can infect insects could be non-pathogenic, or pathogenic to the insects themselves, or to humans and animals. The objective of this systematic review is to provide an overview of the viruses detected in edible insects currently considered for use in food and/or feed in the European Union or appropriate for mass rearing, and to collect information on clinical symptoms in insects and on the vector role of insects themselves. Many different virus species have been detected in edible insect species showing promise for mass production systems. These viruses could be a risk for mass insect rearing systems causing acute high mortality, a drastic decline in growth in juvenile stages and in the reproductive performance of adults. Furthermore, some viruses could pose a risk to human and animal health where insects are used for food and feed.

## 1. Introduction

Edible insects are expected to become an important nutrient source for animals and humans in the Western world in the near future and traditionally hold this status in many tropical countries [1,2]. The reasons why insects are regarded as an alternative source of animal protein are environmental, nutritional and economic [3,4,5,6]. More than 2000 edible insect species are consumed worldwide [7]. The insects most widely consumed by humans belong to the Coleoptera (31%), Lepidoptera (18%), Hymenoptera (14%), Orthoptera (13%) and Hemiptera (10%) [8] orders and can be consumed at different life stages—eggs, larvae, pupae, or adults [4,8,9]. Only a few of the edible species reported in the literature [7] meet the demands of mass rearing systems and industrial activities. Twelve insect species have been reported by EFSA to have the greatest potential to be used as food and feed in the European Union [10]. Edible insects could be harvested seasonally in the wild or reared in controlled environments in most European countries [11]. The different production systems (i.e., industrialized rearing, insect farming, or wild harvesting) by which the edible insects are bred can contribute to differences in their safety [2,11,12,13]. In recent years, due to the novelty and microbial complexity of industrial insect rearing for human consumption, many articles and reviews have explored possible food safety hazards to human and animal health associated with the use of insects for food and feed, including chemical, microbiological and allergenic agents, and prions [10,11,14,15,16,17,18,19,20,21]. In addition to ‘general food hygiene requirements’, the production and marketing of insects as food in the EU is governed by ‘Novel Foods’ legislation—i.e., Regulation (EU) No 2015/2283 [22]; Regulation (EU) 2017/2469 [23]; Regulation (EU) 2017/625 [24]; Regulation (EU) 2019/1381 [25]; Regulation (EU) No 142/2011 [26]; Regulation (EU) 2017/893 [27]. EFSA Scientific Opinion (2015) and adoptions and discussions of the EFSA NDA Panel on Nutrition (2016) are also taken into consideration [10,28].

Both insects collected in Nature and those raised on farms may be infected with pathogenic microorganisms, including bacteria, viruses, fungi, protozoa and other organisms that can affect their safety as food [16,29]. Insects represent the largest group of animals on earth in terms of biodiversity, with an estimated 5.5 million different species [30,31]. This diversity is reflected in a matching range of infecting viruses, which not only positively or negatively affect insect populations, but can also have a major impact on human well-being [32,33]. Insects have been shown to contain a wide variety of viruses up to indicate insects as major reservoirs and vectors of viruses [34,35,36,37,38] and recent papers reveal an enormous diversity in RNA viruses detected in insect viruses [36].

There is an abundant literature on the presence of viruses in insects of economic value or importance to public health, as in the case of silkworms and mosquitoes [39,40,41]. Few studies have been conducted on viruses in edible insects with potential for industrial rearing and concern mainly pathogenic insect viruses, including only some edible insect species [10,16,42]. Insects could harbor a plethora of viruses: (i) microbiota viruses; (ii) viruses pathogenic to insects themselves; (iii) viruses pathogenic to vertebrates, both animal and human. Viruses are part of the normal microbiota of an insect, i.e., its virome, and thus intrinsically associated with insect metabolism, behavior and survival. Albeit under situations of stress, the virome could become pathogenic for the insect [31,43]. Viruses pathogenic to insects can cause a decline in growth and reproductive performance, in addition to disease and mortality. For these reasons, said viruses pose a major concern in insect mass rearing systems where insects are raised at high densities [16,44]. The fact that invertebrate viruses can be transmitted to vertebrates further increases the importance of screening measures for commercially bred prey insects [45]. Viruses pathogenic to vertebrate hosts could be found in insects. While these viruses do not replicate in insects and are not actively transmitted to vertebrates by insect vectors, they could be transmitted passively by insects acting as mechanical vectors [10,46]. This vector capacity highlights the potential of insects produced for food and feed to transmit viral diseases to vertebrates [10]. In some cases, however, insects are replicative vectors of viruses infecting vertebrates. Arboviruses can replicate in their vector, infect vertebrates, and cause severe human (i.e., dengue fever, West Nile disease, Rift Valley fever, hemorrhagic fever, chickungunya fever) and animal (Schmallenberg) diseases [47,48]. To date there is no evidence of these viruses in edible insects.

The focus of this study was on virus species detected to date in edible insect species currently considered for use in food and/or feed in the EU or with characteristics suited to mass rearing systems.

## 2. Materials and Methods

### 2.1. Data Collection Process (Information Sources, Search Strategy, Eligibility Criteria)

For this systematic review, we performed a literature search to identify scientific articles reporting viruses in diverse edible insect species. The information retrieved from the literature conforms to the Preferred Reporting Items for Systematic reviews and Meta-analyses (PRISMA) statement [49].

The insect species included in this review are those considered by EFSA, with the exception of *Bombyx mori*: *Musca domestica*, *Hermetia illucens*, *Tenebrio molitor*, *Zophobas morio*, *Alphitobus diaperinus*, *Galleria mellonella*, *Achroia grisella*, *Acheta domesticus*, *Gryllodes sigillatus*, *Locusta migratora* and *Schistocerca americana* (Table 1). Since the taxonomy and classification of *Zophobas morio* is still unclear and is currently identified as conspecific with *Zophobas atratus* [50], in this review, as in the one by Rumbos and Athanassiou [51], we consider *Z. morio* and *Z. atratus* as one species, referred to as *Z. morio*. However, in order to collect all possible information, both scientific names were used in the string terms.

In addition to the species considered promising by EFSA, we decided to also include: *Tribolium castaneum*, *Gryllus assimilis*, *Gryllus bimaculatus*, and *Schistocerca gregaria* in this review. These species have good potential as edible sources due to their breeding characteristics and/or resistance to viruses. Two of them (*G. assimilis* and *S. gregaria*) have already been listed by Mleck et al. [52] as suitable food species in Europe and other developed countries. Furthermore, *G. assimilis* and *G. bimaculatus* are listed by Weissmann et al. [44] as two of the five most commercially important cricket species worldwide.

Silk and honey are the primary products of the *Bombyx mori* (Silkworm) and *Apis mellifera* (Western honeybee), respectively. Although the edible part of these insects (silkworm pupae and bee brood) is already consumed in many parts of the world and constitutes a promising edible resource, it is not specifically covered in this review because it is only a marginal part of the production system of these insect species [53,54,55].

Relevant studies were searched through two online database repositories, PubMed and Web of Science, using the keywords: “virus*” AND “scientific name of edible insect species” OR “common name of edible insect species” (see Table 1 for the name details). The research was limited to studies published in English up to February 2021. Subsequently, after removing duplicates, only full-text articles were screened, including the grey literature (i.e., materials and research produced by organizations outside traditional commercial or academic publishing and distribution channels); only papers providing original research and data were selected and considered for this review (eligibility assessment). Relevant papers and reviews were also manually cross checked to identify further references, which have been identified as “other sources” in Table S1 The following data were extracted from selected articles and listed in Table S1: bibliographic details (database source, authors, title, source, year of publication, DOI or any PMID) and name of the edible insect species considered. Information regarding viruses detected in each insect species, grouped by order, is discussed in the text and summarized in table form.

### 2.2. Summary of Extracted Data

**Figure 1:**
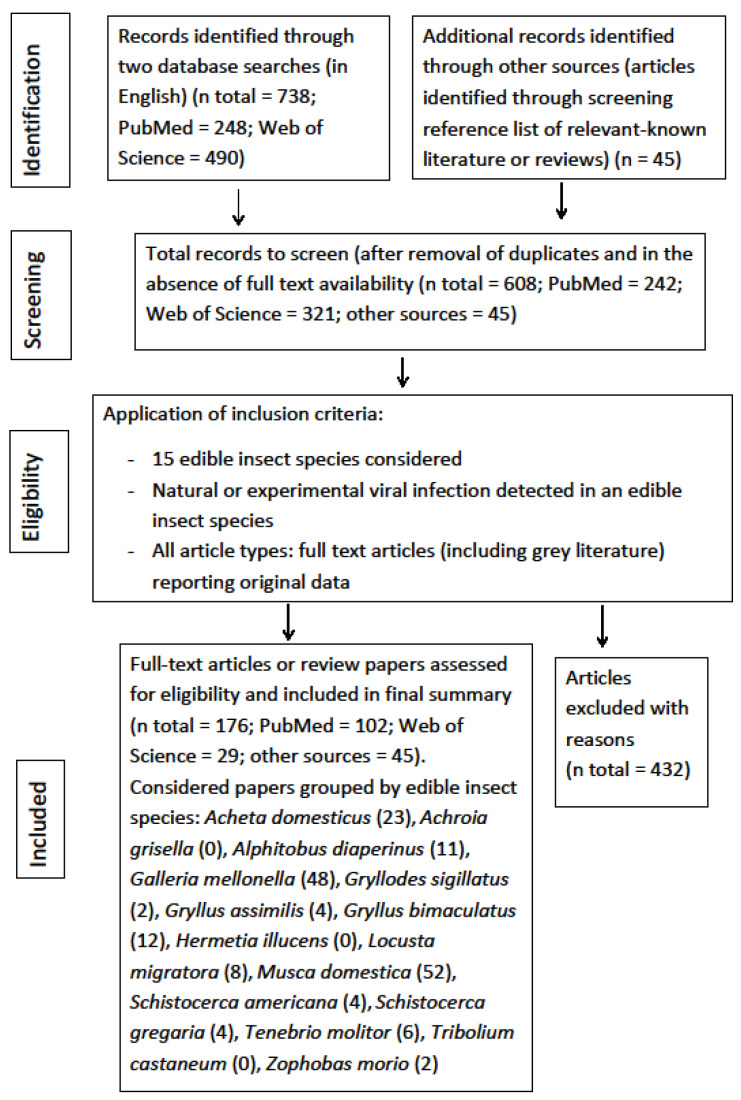
PRISMA flowchart diagram of the selection of eligible studies (literature research up to February 2021).

The four-phase study selection process in the present review is reported in Figure 1. In the initial research a total of 783 studies were identified (PubMed = 248, Web of Science = 490, citations from retrieved articles = 45). Of these, 608 papers were retrieved after removal of duplicates (161) and documents without full text availability (14). Of these, a final 176 articles, fulfilling the inclusion criteria, were identified for this review. The other publications (n = 432) were discarded due to: no virus detected in the insect species considered; no monitoring activity; virus detected in insect species not considered in this review; lack of original data; studies regarding other topics; unclear presentation of data.

## 3. Results

The edible insect species considered in this review are grouped below by order (Coleoptera, Diptera, Lepidoptera and Orthoptera). A specific paragraph has been created for each insect species, describing the main nutritional and productive characteristics, potential for mass rearing systems, and associated viruses. Information on virus characteristics (i.e., family, genus, species, and genomic aspects), insect stage involved during viral infection, type of viral infection (i.e., natural if virus was detected in the wild or experimental if a species of insect was tested for susceptibility to a virus), any detected symptoms or mortality, have been summarized in the tables (Note 1: in this review, chilo iridescent virus (CIV) is used as synonym of invertebrate iridescent virus 6 (IIV-6), the type species of the genus *Iridovirus* within the family *Iridoviridae* [56]; Note 2: within the *Iridoviridae* family, different or novel strains of invertebrate iridescent virus 6 (i.e., invertebrate iridescent virus 29, cricket iridescent virus, *Gryllus bimaculatus* iridescent virus, and lizard-cricket iridescent virus) have been considered separately [57,58,59,60]).

### 3.1. Coleoptera

#### 3.1.1. *Alphitobius diaperinus*

The darkling beetle, *Alphitobius diaperinus*, is one of the most abundant insect pests in commercial poultry production facilities, with cosmopolitan occurrence [61,62,63]. Since 1 July 2017, *A. diaperinus* has been listed as one of the seven insect species authorized to date for use in the large-scale production of processed animal protein (PAP) for aquaculture feeds, in accordance with EU Regulation (EC) No. 2017/893 [27]. For this reason, several papers describing nutritional aspects, breeding facilities, and microbial dynamics during the industrial production cycle [64,65,66,67,68,69] are present in the literature. Although *A. diaperinus* belongs to the species with the greatest potential to be used as food and feed in the EU [10], it could serve as a reservoir and vector for a plethora of pathogenic microorganisms, as bacteria, fungi, coccidia, worms, and tapeworms, and of viruses that cause serious diseases [51,70,71]. It is a mechanical vector of avian viruses belonging to different genuses: Marek’s disease, avian leucosis virus, fowl pox virus (FWPV), infectious bursal disease virus (IBDV), turkey coronavirus (TCV), Newcastle disease viruses, infectious laryngotracheitis virus (ILTV), and reovirus 24 [70,72,73,74,75,76,77,78,79]. These viruses could survive, from a few days to several weeks, inside and on the external surfaces of both adult beetles and larvae [70,75,76,77,79] and could also survive the metamorphosis of this beetle [74].

Only two insect viruses have been found in the lesser mealworm that parasitize honeycombs: black queen cell virus (BQCV) and Israeli acute paralysis virus (IAPV) [80]. These viruses cause severe disease in honeybees, but no signs or symptoms of disease have been noted in *A. diaperinus,* which appears to act only as a vector and reservoir for honeybee viruses [80]. At the present time, neither human foodborne pathogens nor insect viruses pathogenic for this species have been identified [81] but since the analyses involved only a limited number of samples, more studies are needed.

#### 3.1.2. *Tenebrio molitor*

The yellow mealworm *Tenebrio molitor* is one of the largest stored-product beetles, which are widespread across the world [82]. This beetle is increasingly recognized as an optimal alternative and sustainable nutrient source for animal feed and human food due to its protein-rich content and low ecological footprint [65,66,83]. Furthermore, recent studies have shown the ability of mealworm larvae to efficiently degrade polystyrene and plastic waste [84]. Besides being authorized to be used for the production of processed animal protein (PAP) for aquaculture feeds (EU Regulation No. 2017/893) [27], *T. molitor*, in the form of dried larvae, has recently been declared a safe novel food pursuant to Regulation (EU) No. 2015/2283 [22]. *T. molitor* can be infected by or harbor parasites, entomopathogenic fungi, and viruses which reduce mealworm survival or reproductive success [82]. To date, only three insect viruses, belonging to the *Iridovirus* and *Densovirus* genera, were isolated from or tested against *T. molitor* and in one paper this insect was used as an animal model. To date, no foodborne viruses have been detected in industrially reared mealworm larvae [81].

Two species of *Iridovirus* can cause disease leading to death in *T. molitor*: invertebrate iridescent virus 6 (IIV-6) and invertebrate iridescent virus 29 (IIV-29). IIV-6 causes paralysis in *T. molitor* larvae 3 days after virus infection [85] and then death; the color of infected larvae appeared to be darkened only after death. IIV-29, a tentative species of the *Iridovirus* genus [59], rarely encountered in mealworm larvae, produces a particular bluish iridescence in pupae and adults, causing important mortality in *T. molitor* pupae [86,87] within a few days. The third species of virus detected in healthy *T. molitor* larvae is *Acheta domesticus* densovirus (AdDNV) [88]. Mealworms are not considered to be densovirus hosts but when associated with cricket colonies in the same insect farm, they could mechanically transmit AdDNV via their body surface or in the gut [22,88]. *T. molitor* was used as a bioassay animal for *Penaeus merguiensis* densovirus (PmergDNV), which is particularly relevant for aquaculture since it causes disease in crustaceans [89], but it did not appear to be an optimal model for studying this virus.

#### 3.1.3. *Zophobas morio*

*Zophobas morio*, also known as the super worm, is a large beetle originating in South and Central America and introduced into other regions of Europe and Asia in recent years [50,90,91]. *Z. morio* has been used as a protein source for small pets such as birds, reptiles, and small mammals [92], but has the potential to become a promising nutrient source for food and feed [51]. Over the last decade the literature on *Z. morio* as a nutrient source for food, livestock animal feed, and aquaculture has increased considerably [51,93,94]. To date, *Z. morio* is not listed in Regulation (EU) No 2017/893 and is therefore not officially authorized for inclusion in aquafeeds in the EU [27]. The main strengths of this species are: large size compared to *T. molitor*, density independent cannibalism [95], increasing size and weight, formation of supernumerary larval instars that do not pupate until death in crowded conditions [96], high dependence on isolation for metamorphosis onset [97,98].

Little information is available on viral diseases in the super mealworm, with only two reports available to date on virus infection in *Z. morio* larvae [99,100]. The first refers to the collapse of a *Z. morio* colony in Hungary, where sudden large losses (80–90%) of *Z. morio* larvae were observed by a breeder in Budapest. Larvae displayed less activity, unusual behavior, and failed to pupate. Diagnostic methods detected densovirus in colony larvae but no further identification was performed [99]. Tokarev and colleagues [100] reported isolation of a densovirus in *Z. morio* larvae cultured in Russia; in one month, middle-aged larvae developed symptoms of acute disease affecting the locomotion system, inducing swirling, rolling and chaotic wandering of larvae, and within several days, up to 90–100% of the colony had died. Prior to death the larvae showed no morphological changes but after death the cadavers blackened quickly, were more intensively stained, and the inner content was partially liquefied. Through biomolecular techniques the virus was attributed to *Densoviridae*, with maximal similarity to two *Blatella germanica* densovirus-like isolates from the bat [100]. One of these latter viruses could develop within vertebrate tissue [101]. Thus, the hazard of vertebrate host infection with insect densoviruses cannot be excluded [100]. 

Viruses detected in coleopteran edible species are listed in Table 2.

### 3.2. Orthoptera

#### 3.2.1. *Acheta domesticus*

The European house cricket, *Acheta domesticus*, is currently one of the most widely farmed insects, particularly in North America and Europe, and constitutes a thriving pet/reptile feeder insect market worldwide. This cricket also represents an emerging, vibrant insect-based food industry due to its high protein content (about 70% by dry weight), its short life cycle (around 5 weeks), and its prolificacy (females lay more than 1500 eggs) [2,102]. Nutritional and food safety aspects as well as the risk profile of this cricket were extensively studied in recent years [35,103,104,105,106]. At the present time, *A. domesticus* has been authorized for use as processed animal protein (PAP) for aquafeeds (EU Regulation 2017/893) [27].

Viruses affecting this species of cricket have been reported in several papers [16,35,42]. In a recent investigation, five different virus species were detected in commercially reared house crickets from different Swedish retailers [107]. At the present time, the reported viruses consist of insect viruses but no human foodborne viruses have been identified in this specimen [81]. Today, the main virus affecting *A. domesticus* industry is *Acheta domesticus* densovirus, which is able to decimate commercial mass rearings in just a few days, leading to a fall in production and even extinction of local cricket populations [44,88,108,109,110]. This virus was first isolated from diseased *A. domesticus* from a Swiss commercial mass rearing facility in 1977 [111]. In North America, the first report of a densovirus disease in crickets was in 1991 [108] in a small epidemic outbreak. Almost twenty years later, severe outbreaks were observed in commercial facilities in Canada and the United States, causing an acute crisis in the pet food industry [88]. Symptoms of infection include a loss of consistency, smaller dimensions, malnutrition (i.e., absence of contraction and completely empty digestive caeca), inhibited growth, reduced fecundity, increasing sluggishness, less activity, and lower jumping [88,108,109]. Crickets have been observed to lie on their backs paralyzed for several days prior to succumbing to viremia. The highest mortality, of up to 100%, is observed in the last larval stage and in young adults [88,109]. AdDNV positive tissues included the fat body, midgut, hypodermis, and Malpighian tubules [88].

Other viruses observed in cricket facilities are *Acheta domesticus* mini ambidensovirus (AdMADV), a new ambisense densovirus, and *Acheta domesticus* volvovirus (AdVVV), a single-stranded, circular DNA virus; but no further information is currently available on these viruses [112,113,114]. A new iflavirus, *Acheta domesticus* iflavirus (AdIV), has recently been isolated from both wild and commercially reared *A. domesticus* [107,115]. *A. domesticus* is highly susceptible to both IIV-6 and its novel strain, cricket iridovirus (CrIV) [58]. CrIV was isolated in 1996 from *A. domesticus* nymphs and adults from colonies of a commercial cricket producer in Europe. This virus, transmitted orally, led to unusual mortalities as well as greatly reduced fecundity and life span [116]. The fat body was strikingly hypertrophied and, on dissection, displayed a bluish iridescence, which is a typical sign of an iridovirus infection [116].

Cricket paralysis virus (CrPV) was isolated from *A. domesticus* from dead crickets of a commercial cricket farm in the United States of America [117,118]. *A. domesticus* is an omnivorous scavenger which could ingest virus-killed insects, thereby simply becoming a mechanical vector of other insect viruses, as *Autographa californica* multiple nucleopolyhedrovirus (AcMNPV) and two recombinant strains (AcMNPV.AaIT and AcJHE.SG AcMNPV), bait-cricket virus (BCV), and *Solenopsis invicta* virus 3 (SINV-3) [119,120,121,122]. The house cricket has been used as an alternative bioassay model (i.e., in aquaculture) for viral pathogens, with promising results [89,123,124].

#### 3.2.2. *Gryllodes sigillatus*

The banded cricket (or tropical or Indian house cricket), *Gryllodes sigillatus,* is a cosmopolitan cricket that lives in close association with human dwellings [44]. Little information is available on its biology, ecology, rearing, and processing requirements [125,126] but it is one of the species authorized for use for the production of PAP in aquaculture feed (EU Regulation No. 2017/893) [27]. *G. sigillatus* is sold in both US and European pet food stores [44] and its nutritional aspects, functional properties, and microbiological characteristics have recently been studied [125,127,128]. To date only *Acheta domesticus* densovirus (AdDNV) has been detected in *G. sigillatus* but it is less susceptible to AdDNV compared to other orthopteran species [44,81].

#### 3.2.3. *Gryllus assimilis*

The Jamaican field cricket, *Gryllus assimilis*, was first described from Jamaica and is widespread in the West Indies, Brazil, Central America, Mexico, and in five of the southernmost U.S. States [44]. This cricket is used for animal feed in Brazil, is the third orthopteran species authorized for PAP for aquafeeds (EU Regulation 2017/893) [27], but limited literature exists on its application in food [94]. To date, three different insect viruses have been detected in *G. assimilis*: cricket iridovirus (CrIV), *Acheta domesticus* densovirus (AdDNV), and a new volvolovirus named *Acheta domesticus* volvolovirus (AdVVV). *G. assimilis*, especially its first instars, is highly susceptible to CrIV [58], but usually seems to be infected with AdDNV to a much lower degree [44]. Furthermore, AdDNV-exposed *G. assimilis*, reared to adulthood, were reported to produce large numbers of eggs that hatched and developed without displaying signs of virus infection [88]. The novel volvovirus responsible for mass house cricket die-offs in America [112] has been isolated also in *G. assimilis* in Japan but little is known about it [113].

#### 3.2.4. *Gryllus bimaculatus*

Commonly called the two spotted cricket, *Gryllus bimaculatus* is apparently the most widely distributed *Gryllus* species and is found at the tip of South Africa, in northern Europe, and as far east as Thailand [129]. *G. bimaculatus* is commonly consumed as food in different parts of the world [130,131]. Numerous studies have investigated its dietary requirements for rearing procedures [132,133], and its utilization as a biowaste consumer [134] and food and feed resource [130,135,136,137].

To date seven different viral pathogens have been described in reared *G. bimaculatus* [34,35,42] belonging to *Iridovirus*, *Densovirus*, *Nudivirus* and *Cripavirus*. *G. bimaculatus* seems to be highly susceptible to iridovirus but resistant to densovirus [58,88]. Within the *Iridoviridae* family, invertebrate iridescent virus (IIV-6) and two variants or novel strains of IIV-6—*Gryllus bimaculatus* iridescent virus (GbIV) and cricket iridovirus (CrIV) could seriously damage *G. bimaculatus* mass production, causing colony collapse in two weeks [58,116]. Invertebrate iridescent virus type 6 (IIV-6) is reported to be responsible for bluish iridescence in the fat body, malformations i.e., distorted development of the wings in patently infected crickets, and inability to complete ecdysis [60]. Cricket iridovirus (CrIV), instead, caused fatal infections in this cricket species with mortality potentially exceeding 90% [116]; third instars had overt signs and symptoms of virus infection, as swollen abdomens and a striking sluggishness. *Gryllus bimaculatus* iridescent virus (GbIV) caused the death of all individuals of a colony by 14 days, showing clinically apparent behavior as apathy, ataxia, and disorientation [57]. The fact that neither CrIV nor GbIV have been observed in the ovarian cells of the insect host, suggests that transovarial transmission of these viral infections is unlikely [57] and could help in viral disease management in an epidemic spot. An invertebrate iridovirus was isolated from several tissues of a high-casqued chameleon (Cham_IIV). The pathogenicity of this isolate for *G. bimaculatus* was tested, revealing mortality rates of between 20% and 35%, and the virus was then re-isolated from several fat-body samples of the cricket [45]. These findings support the hypothesis that IIV from insects can infect reptiles and amphibians via the insects on which they feed [45,60,138,139]. Specifically, GbIV have been detected in three different species of reptiles [138], while lizard-cricket iridovirus (Liz_CrIV), a new strain of CrIV, has been isolated from crickets, reptiles and amphibians [60].

*Gryllus bimaculatus* nudivirus (GbNV) [140,141,142] can cause disease and mortality in *G. bimaculatus* nymphs but has a chronic course in adult specimens. Infections are reported to occur primarily during nymphal development, especially by cannibalistic feeding on moribund or dead specimens [140]. Affected crickets are smaller and sometimes get crippled. They may molt repeatedly while becoming progressively uncoordinated and show lethargic behavior till they finally die, within weeks, often only in the final instar stage. In the advanced stage of disease, crickets are often strikingly swollen, and harbor an enormous amount of viscous, milky opalescent hemolymph, giving them a sticky consistency [140]. As regards *Acheta dometicus* densovirus (AdDNV), while testing positive at the lowest dilution, *G. bimaculatus* so far appears resistant [44,88]. Cricket paralysis virus (CrPV), multiplying in the two spotted crickets, causes paralysis of the hind legs, especially in young instars, and death in 8 to 9 days with a very high mortality [117,118,143].

#### 3.2.5. *Locusta migratoria*

The African migratory locust *Locusta migratoria* is one of the insects responsible for crop devastation in certain developing countries and among the most economically important locusts [58,144]. Viruses detected in *L. migratoria* belong to four families, i.e., *Poxviridae*, *Baculoviridae*, *Iridoviridae*, and *Reoviridae* [58,116,144,145,146,147,148,149,150]. *L. migratoria* is susceptible, to varying degrees, to entomopoxviruses (EPVs), isolated from different orthopteran species [144,145,146], and capable of causing disease and mass mortality in its natural population [144]. Usually the immature specimens (1st to 4th instars) are the most affected and the disease leads to heavy mortalities, despite following a chronic course [145,146]. For example, the mortality of *L. migratoria* nymphs fed with entomopoxvirus occlusion bodies isolated from *Melanoplus sanguinipes* reached 90% in the 60 days after virus inoculation [146] or caused entire colony death before maturation [145]. Arphia conspersa entomopoxvirus displays lower pathogenicity, causing a mortality level of approximately 68% of the colony population within 60 days after virus inoculation [147].

*L. migratoria* can be infected with nucleolyhedrovirus of *Spodoptera littoralis* (SINPV-type B), a Lepidopteran species [148,149]. This virus was reported to be involved in a disease outbreak in immature *L. migratoria,* showing a disease pattern termed ‘dark cheeks’. However, the viral amounts in the infected locusts were very low and the role of the virus in generating the observed disease remained ambiguous [148]. A subsequent experiment revealed the slow, gradual disappearance over time of viral DNA post infection while no signs of disease were observed in the infected locusts, suggesting that SINPV does not multiply in locusts and is not therefore pathogenic to *L. migratoria* instars [149]. A significant increase in mortality in *L. migratoria* was recorded during cytoplasmic polyhedrosis virus (CPV) infection [150]; to date, there is no further information about this virus. Young nymphs of *L. migratoria* were heavily infected by the two virus isolates, invertebrate iridescent virus (IIV-6) and cricket iridovirus (CrIV), with mortality as high as 100% [58]. Iridoviruses can be transmitted perorally to *L. migratoria*, causing characteristic symptoms and fatal disease [116].

#### 3.2.6. *Schistocerca gregaria*

The desert locust *Schistocerca gregaria*, due its high reproductive potential, represents another major pest in agriculture and, as other locust species, has been extensively studied in previous years [58,144]. In 1968, a virus called “Schistocerca virus” was detected in 5th instars of *S. gregaria,* showing signs of inactivity and a high death rate [151]. *Spodoptera littoralis* nucleopolyhedrovirus (SINPV) was reported to be involved in a disease outbreak in immature *S. gregaria* [149]. Entomopoxvirus in wild specimens of *S. gregaria* was detected by Purrini and Rohde [152]. Younger instars of *S. gregaria* (L1–L3) could also be heavily infected by the two *Iridovirus:* invertebrate iridescent virus (IIV-6) and cricket iridovirus (CrIV) [58].

#### 3.2.7. *Schistocerca americana*

Limited information exists on *Schistocerca americana*, commonly known as the American grasshopper [153]. In 1966, Henry and Jutila [154] isolated a polyhedrosis virus from a grasshopper, *M. sanguinipes*, and infected *S. americana*. The latter species proved only slightly susceptible: after a latent period of about 12 days, infected specimens exhibited general torpor, a decreased rate of development, and eventual death. The fat body—the only tissue invaded—became generally hypertrophic, changing from its normal glossy yellow to a fluffy gray. Examination of sectioned tissues revealed that most of the fat-body cells were filled with large numbers of polyhedral bodies [154]. Field-collected *S. americana* specimens, showing symptoms and signs of disease, were found to be infected with Melanoplus sunguinipes entomopoxvirus. Placed in a rearing facility, these specimens spread the virus to other grasshopper species through horizontal transmission, apparently by consumption of infected cadavers [147,155]. The crystalline-array virus (CAV), a small RNA virus belonging to the picornavirus group, causes death or morbidity of 5th instars of *S. americana* 6 days after intrathoracic inoculation [156,157]. 

Viruses detected in orthopteran edible species are listed in Table 3.

### 3.3. Diptera

#### 3.3.1. *Hermetia illucens*

The Black Soldier Fly (BSF), *Hermetia illucens*, is a saprophytic insect, which currently has a cosmopolitan distribution in tropical and temperate areas [159,160]. *H. illucens* is one of the most promising insect species for food and feed production, efficient bioconversion of food waste, and biodiesel and fertilizer production [161,162,163,164,165]. The main aspects that make the BSF easy to rear and a suitable tool to valorize waste and sustainable animal feed or human food sources are: (i) the diversity of the substrates they can process and the efficiency with which they do so may be highest among the flies [166]; (ii) their feed conversion ratios are known to be superior to both crickets and mealworms [3]; (iii) prepupae instinctively leave the substrate and move to a high, clean place, a behavior called “self-harvesting” which removes an otherwise labor-intensive step from their farming [167].

In recent years, economic interest in this species has grown yielding abundant scientific literature on its behavior, rearing, nutritional value, and industrial applications [164,168,169,170,171,172,173]. According to current knowledge, no viruses have been reported in this species, either at the larval stage or in the adult, despite it being a scavenger and its life cycle being associated with polluted environments [174]. In one paper, BSF larvae proved capable of efficient microbial load reduction in the substrate contaminated with different pathogen microrganisms (*Salmonella* spp., Orthoreovirus, Mastadenovirus, Teschovirus), but the analyses were performed only on the substrate and not on the larvae [175].

#### 3.3.2. *Musca domestica*

The housefly, *Musca domestica*, distributed worldwide, is the most common species of fly, living in close association with humans and domestic animals, making it one of the most highly studied insect pests [176,177]. Besides being a source of irritation and spoiling food, the house fly acts as a vector for many medical and veterinary pathogens [178]. The ability of *M. domestica* to reproduce quickly could be exploited to produce larvae as a source of protein for animal feed [179,180,181,182]. Moreover, like *H. illucens*, larvae could grow in different substrates making *M. domestica* a promising insect for organic waste degradation [183,184,185,186]. An abundant literature already exists in relation to mass rearing systems for *M. domestica* [180,187].

The housefly is known to carry more than 130 pathogens, including bacteria, viruses, fungi and parasites, some of which can cause serious, life-threatening diseases in humans and animals, or diseases in flies themselves [176,177]. *M. domestica* can harbor several viruses pathogenic for both humans and livestock but, to date, only two insect viruses that are pathogenic for the fly itself have been detected [188,189,190,191]. Flies have been shown to mechanically transmit pathogens via their mouthparts, vomit, faeces, and whole body surface [176,190]. Contrary to *H. illucens*, *M. domestica* adults are synanthropic and therefore any pathogens being carried could easily be transmitted to humans, animals, and other flies. Adult flies act only as mechanical carriers of human viruses after contamination by infected human fecal material [191,192,193]. These potential passive contaminators are capable of carrying and depositing the virus at a considerable distance from the point of original contamination. Human viral pathogens mechanically transmitted by adult flies include: coxsackieviruses, enteroviruses, rotaviruses, and poliomyelitis virus [190,191,192,193,194,195,196,197]. In addition, one study demonstrated the ability of the housefly to carry the Ebola virus in laboratory experiments but the role of the common fly in transmission of the virus remains to be confirmed [198].

The main virus affecting adult *M. domestica* is *Musca domestica* salivary gland hypertrophy virus (MdSGHV). It was isolated in 1993 [199] and then extensively studied. MdSGHV has been detected in housefly samples from North America, Europe, Asia, the Caribbean, and the southwestern Pacific [199]. Populations of *M. domestica* (only adults) are naturally infected with MdSGHV but its incidence varies widely among farms and at different times of the year [200], with the highest prevalence in summertime [200,201]. The virus causes symptomatic salivary gland hypertrophy (both nuclear and cellular), with a characteristic white-blue color, in both genders of *M. domestica* flies (although males seem more affected), in addition to suppressing ovarian development in infected females, inhibiting egg production and resulting in female sterility [189,202,203,204,205]. Male reproductive performance is also affected by virus infection [203]. Since sexual and vertical transmission have been ruled out, this virus only spreads horizontally [201,202,203,204]. MdSGHV, produced in the gland cells, is continuously shed during feeding, resulting in contamination of food material; the deposition of oral secretions and excreta onto a shared food substrate is the main route of natural MdSGHV transmission among adult house flies [199,206]. Typically, in natural populations, this virus has not been observed to cause the widespread epizootics characteristic of other insect viruses [200] and the introduction of MdSGHV-infected flies into confined populations does not produce epizootics but results in a persistent, albeit declining, prevalence of viral infection [207]. The virus was able to infect > 50% of newly eclosed adults whereas older adults were highly resistant to infection (0–5%) [199]. Infected male and female flies consumed significantly lower quantities of protein and sucrose than control flies; this suggests that MdSGHV has a negative consumption effect (e.g., hunger, starvation) on its host [203,208,209,210].

The second virus causing disease and mortality in *M. domestica* adults is a reovirus, detected by Moussa in 1978 and now named idnoreovirus 3 (Idno-3) [188]. This virus, multiplying in the hemocytes of infected flies, produced morphological alterations (i.e., swollen abdomen, enlarged, brownish midgut) and motor dysfunctions such as trembling of wings and legs and total paralysis. Mortality began within the first 24 h after emergence of adults, causing colony collapse in 10 days [188,211]. No mortality was observed in early larvae, but a few dead final instar larvae were found.

*M. domestica* has also been reported to mechanically transmit several types of viral pathogens to livestock including: avian influenza virus (AIV), both high and low pathogenic strains [46,212,213,214,215,216], turkey coronavirus (TCV) [217], Newcastle disease virus (NDV) [218,219,220,221,222], reticuloendotheliosis virus (REV) [223], porcine reproductive and respiratory syndrome virus (PRRSV) [224,225,226,227,228], porcine circovirus genotype 2 (PCV2b) [229], porcine epidemic diarrhea virus (PEDV) [230], African swine fever virus (ASF) [231,232], Aujeszky’s virus (PRV-1) [233], senecavirus A (SVA) [234], Rift Valley fever virus (RVFV) [235], Aleutian mink disease virus (AMDV) [236,237], and lumpy skin disease (LSDV) [238,239].Viruses detected in dipteran edible species are listed in Table 4.

### 3.4. Lepidoptera

#### 3.4.1. *Achroia grisella*

The lesser wax moth, *Achroia grisella*, is a species closely related to *Galleria mellonella* [241]. As compared to the greater wax moth, the lesser wax moth is less destructive and less common [242,243]. To date in the literature no virus has been detected in this species.

#### 3.4.2. *Galleria mellonella*

The greater wax moth (GWM), *G. mellonella*, is a ubiquitous pest of field-based honeybee colonies and stored combs due to the destructive feeding habit of its larvae [241,244]. Recently, this pest has garnered greater attention as a promising food and feed resource and as an infection model organism. *G. mellonella* has been available as pet food and bait for many years in several European countries and in the USA [42]. This moth can be easily reared, standardized protocols already exist for its breeding and diets [245,246,247,248,249], and it has high nutritional value [19]. *G. mellonella* is a reliable model organism to assay pathogenicity of human, animal and insect pathogens (i.e., bacteria, fungi and viruses) as well as to test the effectiveness and toxicity of antimicrobial compounds [250,251,252,253]. A few studies have collected information on viral diseases involving both insect and mammal pathogenic viruses in *G. mellonella* [42,250], but viral infections have been detected or tested in larval stages only. Regarding animal pathogens, only one paper has to date studied the immune response of *G. mellonella* infected with bovine herpes simplex virus-1 (BHSV-1) [254]. In the wax moth larvae, BHSV-1 stimulates both cellular and humoral immune response in a dose-dependent manner in *G. mellonella* larvae, but no mortality was detected [254].

Nodamura virus, an insect picornavirus that can also infect vertebrates, is able to infect and kill greater wax moth larvae [255,256]. The infected larvae manifest paralysis of the last 5 or 6 segments four to six days after inoculation of the virus. The paralysis progressively spreads to the other segments, leading to death 15 to 20 days after infection [256]. Replication of Nodamura virus takes place in the interfibrillar spaces of the sarcoplasm in close association with the mitochondria in the infected muscles, causing aggregation and shape modification of numerous mitochondria (elongation, interdigitation, and vesiculation) [257]. At a later stage, degenerated, dilated mitochondria show clear assembling of virus particles on their outer membrane and occasionally on some inner membranes [257].

Many insect viruses have been detected in *G. mellonella* larvae. They belong to *Densovirus*, *Iridovirus*, *Baculoviruses*, *Cripavirus* and *Triatovirus* genera and can lead to patent and asymptomatic infections or severe symptoms and mass mortality. For example, *Galleria mellonella* densovirus (GmDV) can cause the death of the entire colony while *Acheta domesticus* densovirus (AdDNV) cannot even infect the colony [90,258]. *Galleria mellonella* densovirus (GmDV) is the most highly studied and described densovirus in this moth species [42,259]. GmDV is highly virulent for young larvae, the most susceptible being third instar larvae, where it seems to replicate more successfully compared to older instars [258]. The mortality rate in infected larvae can reach up to 100% in an average period of 10 days. Infection of prepupae also causes abnormal or absent pupation with no adults emerging [258]. Nucleopolyhedrovirus has been isolated from both *G. mellonella* specimens and other insect species (i.e., *B. mori*, *H. virescens*, *M. franconicum*). All said viral isolates have been shown to be pathogenic especially for *G. mellonella* larvae [260,261,262,263,264,265].

*Galleria mellonella* nucleopolyhedrovirus (GmNPV) is highly pathogenic for *G. mellonella,* particularly in the preimaginal stages (third instars) [262,265,266,267]. This virus multiplies in insect cells causing hypertrophy of the nuclei with the formation of virus-containing inclusion bodies (polyhedra), while non-occluded virus (NOV) particles can be seen in diseased tissues. After 8–10 days, cell destruction causes the release of polyhedra in the infected tissue [268]. During viral infection, the amount of potassium dramatically drops causing severe acidosis, and higher sulfur levels are present compared to healty larvae [261]. *G. mellonella* larvae infected with *Bombyx mori* nucleopolyhedrovirus (BmNPV) ceased to feed and began to wilt after 17 days. Mortality occurred during between 19 and 27 days. Some infected insects did not die until entering the pupal stage [263]. The *Malacosoma franconicum* nucleopolyhedrovirus (ManeNPV-T3) caused 25% mortality in third instars of *G. mellonella* [264].

*G. mellonelIa* is highly permissive, with different levels of magnitude, to many *Iridoviruses* (IVs) isolated from insect species belonging to different orders [50,61,88,159,269,270,271,272,273]. However, in some cases, IIVs causing patent disease in certain insect species do not replicate in *G. mellonella* larvae [274]. Invertebrate iridescent viruses cause pupal malformation and patches of translucent cuticle in the area between the abdomen and ventral thorax through which the iridescent color of the insect can be viewed [270,271]. The viruses cause patent infection with a prevalence of up to 75%; in the case of high-level infection, all insects became patently infected; mortality started 10 days after infection and larvae failed to reach adulthood [271,275]. Cricket iridovirus (CrIV) caused fatal infections in larvae of the greater wax moth [61,116]. Tipula iridescent virus (TIV) can infect and multiply in hemocytes of *G. mellonella* larvae [269,276,277,278,279,280]. Tipula iridescent virus was consistently infectious and caused complete mortality among *G. mellonella*, with the average period to death occurring at about 14 days after injection [269]. Wax-moth larvae inoculated with TIV and reared at 23–25 °C died from virus infection, but at 30 °C and higher temperatures, most of them survived to become adults [281]. Apis cerana iridescent virus (AIV) failed to multiply in *G. mellonella* larvae [282]. Seriscethis iridescent virus (SIV), isolated from *Sericesthis pruinosa*, develops in *G. mellonella* plasmatocytes and adipohemocytes leading to visible alteration (hypertrophy) in these cells within 24 h of virus inoculation [283,284,285]. Cricket paralysis virus caused mortality in *G. mellonella* larvae [286]; specifically, mortality occurred within 5 days in infected penultimate instar larvae [287]. Honeybee viruses, Israeli acute paralysis virus (IAPV), and black queen cell virus (BQCV), have been detected in wax moth larvae but no data are available on their possible pathogenicity in this species [288].

Mycoviruses are a specific group of viruses that naturally infect and replicate in fungi and are able to alter fungal growth. Accordingly, a lower initial concentration of spores can still become lethal post infection. Infection of *A. fumigatus* with A78 mycovirus caused a significant increase in radial growth and virulence in a moth model [289]. Other reports of viruses detected in *G. mellonella* concern a small spherical virus, Galleria free virus (GFV), isolated from *G. mellonella* [290]. The *Galleria mellonella* cell line virus (GmclV), apparently persistently infects the *G. mellonella* cell line (GmclV) and can be efficiently induced to replicate by the introduction of other insect viruses, causing complete death of the colony in 5 days [51]. The last two viruses reported in *G. mellonella*—Pariacoto virus and *Heteronychus arator* virus—show striking similarities to Nodamura virus [291]. Pariaocto virus, isolated in Peru from the Southern armyworm (*Spodoptera eridania*), is able to multiply in *G. mellonella* larvae [292,293]. Fourteen days post infection, Heteronychus arator virus renders *G. mellonella* larvae inactive, flaccid, unresponsive to touch, and paralyzed. Mortality reaches 50% in 20 days after infection [294] and the midgut seems to be the primary target tissue.

Viruses detected in lepidopteran edible species are listed in Table 5.

## 4. Prevention, Control and Management of Viruses in Edible Insect Mass-Rearing Facilities

At present there is no treatment for viral infections in edible insects and therefore prevention and control measures are pivotal for insect mass rearing systems. Mass rearing strategies must focus on defining and standardizing Good Farming Practices (GFP) [35]. First of all, avoiding the entry of insect viruses into highly intensive rearing facilities must be considered paramount. For this purpose, regular analyses must be performed on products entering the rearing systems, including both feeding substrates and new individuals for inbreeding avoidance. Since horizontal (oral) transmission is the main route for the spread of insect viruses, attention should be paid when new breeders (from the same or different facilities), are added to older ones in the reproduction sector, or when eggs and deposition substrates are introduced into the production sector. High attention should be paid also to operator handling hygiene. In addition, stressful conditions (i.e., feeding imbalances or high density) must be avoided to reduce the spread of infection associated with the cannibalistic behavior of certain insect species. It is crucially important in commercial large-scale production to avoid high breeding densities because they increase cannibalism, which in turn enhances the transmission of microbial agents [296]. It is equally necessary to develop efficient protocols that permit early detection of viruses [158]. For example, at the present moment it is possible to perform a qualitative PCR-based detection of AdDV in different substrates, i.e., whole body, body parts or fecal material [44,88,158].

## 5. Discussion

The retrieved literature reveals that many viruses, belonging to 22 different families, have been observed in edible insect species (Table 2, Table 3, Table 4 and Table 5). However, there are no reports at present on virus detection for two species (*A. grisella* and *H. illucens*), while reports on others are sometimes very limited in size or old. Among the insect orders considered, orthopteran seems to be the one most affected by viral pathogens belonging to seven families (i.e., *Dicistoviridae*, *Parvoviridae*, *Nudiviridae*, *Iridoviridae*, *Baculoviridae*, *Poxviridae*, and *Picornaviridae*); the most concerning viruses affecting Orthoptera are species belonging to *Iridoviridae* and *Densoviridae* families. The Lepidoptera order (represented by *G. mellonella*) seems heavily affected by virus species belonging to *Baculoviridae* and *Iridoviridae* families, while *Densoviruses* are rarely reported; other virus species reported to affect *G. mellonella* are member of *Dicistoviridae*, *Parvoviridae* and *Picornaviridae* families. Two iridoviruses and one densovirus are reported to cause mortality in the coleopteran order while only two insect viruses have been described as pathogenic for Diptera.

In the retrieved literature, viruses detected in edible insects can be divided into three major categories: (i) viruses that neither multiply nor cause disease in edible insect species; (ii) viruses that multiply and cause disease and mortality in edible insect species; (iii) viruses that multiply but do not cause disease or decrease performance in edible insect species (asymptomatic). In the first case, those viruses are casually associated with edible insects that act only as a mechanical vector without affecting their productivity or prolificacy (for example: viruses reported from wild caught *M. domestica*). In the other two cases (viruses that multiplied in edible insect species), edible insects act as a biological vector. In the investigated edible insect species, different patterns of viral infection can occur ranging from asymptomatic to highly pathogenic, or even lethal [31]. These distinct outcomes exist in a variable range and can be classified into three main groups namely: acute, persistent, and latent. Acute infections, characterized by high levels of viral replication, are limited in time due to the death of the host or clearance of the virus by the host immune system. Persistent infections, characterized by low viral replication, do not affect host fitness even when they last for longer periods. Abundant covert infections have also been reported from several host insect species [297]. Covertly infected insects appear healthy and the infection is not lethal. Latent infections consist of the presence of a viral genome in the host, without viral particle production, but virus reactivation is possible [31,298].

Viruses that pose the highest risk for the collapse of mass insect rearing systems are those causing acute and high mortality because they can decimate commercial mass farms or entire colonies within a few days [44,88,100,258]. Another threat for edible insect producers are viruses that do not cause mortality but bring about a drastic decline in growth in the juvenile stages and in adult reproductive performance, with total collapse of the colony taking longer [190,204]. Finally, attention should be paid to latent viruses which can reactivate when insects are subject to stressful conditions (i.e., a change in diet or environmental conditions) or concomitant infections, starting with viral particle production and visible effects on reared insects [31,299]. Some viruses affect only the adult stage (MdSGHV in adults of *M. domestica* or Invertebrate iridovirus 29 in *T. molitor* pupae and adults) with no consequences for larvae, representing the valuable edible part, while other viruses affect only larvae (*Galleria mellonella* densovirus in third instar *G. mellonella*). Greater attention must therefore be paid to these viruses in the production sector of insect facilities.

In heterometabolic insects, all stages can be infected by viruses but the juvenile stages are usually the most affected (i.e., cricket iridovirus in nymphs and adults of *A. domesticus*) [58,116]. The same virus could affect different insect species with different degrees of severity. *A. domesticus* is highly susceptible to *Acheta domesticus* densovirus while *G. assimilis* usually seems to be infected with AdDNV to a much lower degree; *G. sigillatus* is less susceptible to AdDNV compared to other orthopteran species and *G. bimaculatus* so far appears resistant [44,47,90]. For this reason, *G. assimilis*, *G. sigillatus,* and *G. bimaculatus* have been proposed as the best replacement crickets to avoid heavy losses in commercial production [44,81]. Besides causing losses in insect mass rearing systems, insect viruses can infect vertebrate hosts [45,63] and the fact that invertebrate viruses may be transmitted to vertebrates further increases the importance of screening measures for commercially produced prey insects [45].

To date, only one article has investigated the presence of foodborne viruses in three species of industrially reared insects for food [81], yielding negative results for the presence of detectable quantities of hepatitis A virus, hepatitis E virus, and norovirus genogroup II. The possibility that human viruses could infect and multiply within edible insects is unclear, but it seems unlikely [35,298]. At the present day, the risk of transmitting foodborne viruses to humans via edible insects (*T. molitor*, *A. diaperinus* and *G. sigillatus*) is considered low. Foodborne viruses could be introduced through rearing substrate or operator handling and transferred beyond primary production. This prompts the need to carry out more studies and experimental infections to produce more evidence of the low safety risk from foodborne viruses.

## 6. Conclusions

The retrieved literature revealed that the number of viruses detected in edible insects is high, with more than 70 species listed and 36 able to cause disease and mortality. Viruses could be more or less species-specific and could infect edible insects at different life stages. Only insect-specific viruses could be a matter of concern in mass-rearing systems as they actively replicate and persist on the target species. Viral infection could have different consequences on mass rearing systems ranging from asymptomatic infection to the entire collapse of the colony. Since to date there is no cure for viral infections in edible insects, preventative measures are the only affordable strategy available. Thus, biosecurity is pivotal for insect mass rearing systems.

To enable edible insects to become a safe nutrient source for animals and humans in the Western world, more investigations are warranted to better understand the effective impact of both insect and vertebrate viruses in industrialized rearing systems.

## Figures and Tables

**Table 1:** List of edible insect species considered in this review.

Order	Family	Genus	Species	Common Name	Stage Consumed	Insects Potentially Suitable for Use in Food and Feed in the EU *
Coleoptera	*Tenebrionidae*	*Tenebrio*	*T. molitor* (Linnaeus, 1758)	Mealworm	larvae	X **
			*T. castaneum* (Herbst, 1797)	Red flour beetle	larvae	
		*Zophobas*	*Z. morio* (Fabricius, 1776)	Super worms	larvae	X
		*Alphitobius*	*A. diaperinus* (Panzer, 1797)	Lesser mealworm	larvae	X **
Diptera	*Muscidae*	*Musca*	*M. domestica* (Linnaeus, 1758)	Common housefly	larvae	X **
	*Stratiomyidae*	*Hermetia*	*H. illucens* (Linnaeus, 1758)	Black soldier fly	larvae	X **
Lepidoptera	*Pyralidae*	*Piraliini*	*G. mellonella* (Linnaeus, 1758)	Greater wax moth	larvae	X
		*Achroia*	*A. grisella* (Fabricius, 1794)	Lesser wax moth	larvae	X
Orthoptera	*Gryllidae*	*Acheta*	*A. domesticus* (Linnaeus, 1758)	House cricket	adult	X **
		*Gryllodes*	*G. sigillatus* (Walker, 1869)	Banded cricket	adult	X **
		*Gryllus*	*G. assimilis* (Fabricius, 1775)	Jamaican field cricket	adult	X **
		*Gryllus*	*G. bimaculatus* (De Geer, 1773)	Two spotted cricket	adult	
	*Acrididae*	*Locusta*	*L. migratora* (Linnaeus, 1758)	African migratory locust	adult	X
		*Schistocerca*	*S. gregaria* (Forskål, 1775)	Desert locust	adult	
		*Schistocerca*	*S. americana* (Drury, 1770)	American grasshopper	adult	X

Legend: * Commission Regulation (EU) No 142/2011; EFSA Scientific opinion, 2015 [10,26]; ** Insect species authorized to be used for the production of processed animal protein (PAP) for aquafeeds in accordance with EU Regulation 2017/893 (7 species) [27].

**Table 2:** Viruses detected in coleopteran edible species.

Coleopteran Species	Virus Family	Virus Genus	Virus Species	Virus Characteristics	Type of Infection	Vector Status	Stage Involved	Symptoms or Mortality	References
*Alphitobus diaperinus*	*Birnaviridae*	*Avibirnavirus*	IBDV	dsRNA	N, E	M	adult	no	[70,73]
	*Coronaviridae*	*Gammacoronavirus*	TCV	dsRNA	E	M	adult	no	[78]
	*Dicistroviridae*	*Triatovirus*	BQCV	ssRNA	N	M	adult	no	[80]
	*Dicistroviridae*	*Aparavirus*	IAPV	ssRNA	N	M	adult	no	[80]
	*Herpesviridae*	*Iltovirus*	ILTV	dsDNA	N	M	adult/larva	no	[80]
	*Paramyxoviridae*	*Avulavirus*	NDV	ssRNA	E	M	adult	no	[75]
	*Poxviridae*	*Avipoxvirus*	FWPV	ssRNA	E	M	adult	no	[75]
	*Reoviridae*	*Orthoreovirus*	AVR	dsRNA	N, E	M	adult/larva	no	[74,76,77]
	*Reoviridae*	*Rotavirus*		dsRNA	E	M	larva	no	[76]
	*Retroviridae*	*Alpharetrovirus*	ALV	ssRNA	N	M	adult	no	[72]
*Tenebrio molitor*	*Iridoviridae*	*Iridovirus*	IIV-6	dsDNA	E	B	larva	yes	[85]
	*Iridoviridae*	*Iridovirus*	IIV-29	dsDNA	N, E	B	larva	yes	[86,87]
	*Parvoviridae*	*Densovirus*	AdDNV	ssDNA	N	M	larva	no	[88]
	*Parvoviridae*	*Densovirus*	PmergDNV	ssDNA	B	Bi	larva	yes	[89]
*Zophobas morio*	*Parvoviridae*	*Densovirus*	ZbDNV	ssDNA	N, E	B	larva	yes	[100]
	*Parvoviridae*	*Densovirus*		ssDNA	N	nd	larva	yes	[99]

Legend: infectious bursal disease virus (IBDV); turkey coronavirus (TCV); black queen cell virus (BQCV); Israeli acute paralysis virus (IAPV); infectious laryngotracheitis virus (ILTV); Newcastle disease virus (NDV); fowl pox virus (FWPV); avian reovirus (AVR); avian leucosis virus (ALV); invertebrate iridescent viruses 6 (IIV6); invertebrate iridescent viruses 29 (IIV29); *Acheta domesticus* densovirus (AdDNV); Penaeus merguiensis densovirus (PmergDNV); *Zophobas morio* densovirus (ZbDNV); single strain DNA (ssDNA); double strain DNA (dsDNA); single strain RNA (ssRNA); double strain RNA (dsRNA); natural infection (N); experimental infection (E); bioassay animal (Bi); mechanical vector (M); biological vector (B); not determined (nd).

**Table 3:** Viruses detected in orthopteran edible species.

Orthopteran Species	Virus Family	Virus Genus	Virus Species	Virus Characteristics	Type of Infection	Vector Status	Stage Involved	Symptoms or Mortality	References
*Acheta domesticus*	*Baculoviridae*	*Alphabaculovirus*	AcMNPV, AcMNPV.AaIT, AcJHE.SG AcMNPV	dsDNA	E	M	adult	no	[119,120]
	*Dicistroviridae*	*Cripavirus*	BCV	ssRNA	N	M	adult	no	[121]
	*Dicistroviridae*	*Cripavirus*	CrPV	ssRNA	N	B	nd	yes	[117,143]
	*Iflaviridae*	*Iflavirus*	AdIV	ssRNA	N	B	adult/nymph	no	[115]
	*Iflaviridae*	*Iflavirus*	Chequa iflavirus	ssRNA	E	Bi	nd	no	[124]
	*Iridoviridae*	*Iridovirus*	CrIV	dsDNA	N, E	B	adult/nymph	yes	[58,116]
	*Iridoviridae*	*Iridovirus*	IIV-6	dsDNA	E	B	nymph	yes	[107]
	nd	nd	AdVVV	ssDNA	N	nd	nd	nd	[107,112,113]
	*Parvoviridae*	nd	AdMADV	ssDNA	N	nd	nd	nd	[114]
	*Parvoviridae*	*Densovirus*	AdDNV	ssDNA	N, E	B	adult/nymph	yes	[44,88,107,108,109,110,158]
	*Parvoviridae*	*Densovirus*	PmergDNV	ssDNA	E	Bi	nd	yes	[89,123]
	*Phenuiviridae*	nd	Bunya-like virus	ssRNA	E	Bi	nd	no	[124]
	*Solinviviridae*	*Invictavirus*	SINV-3	ssRNA	E	M	adult/nymph	no	[122]
	*Nudiviridae*	*Alphanudivirus*	GbNV	dsDNA	N	B	adult/nymph	yes	[107]
*Gryllodes sigillatus*	*Parvoviridae*	*Densovirus*	AdDNV	ssDNA	E	B	nd	nd	[44]
*Gryllus assimilis*	*Iridoviridae*	*Iridovirus*	CrIV	dsDNA		B	nymph	nd	[58]
	nd	nd	AdVVV	ssDNA	N	nd	nd	nd	[113]
	*Parvoviridae*	*Densovirus*	AdDNV	ssDNA	N, E	resistant			[44,88]
*Gryllus bimaculatus*	*Dicistroviridae*	*Cripavirus*	CrPV	ssRNA	N	B	nymph	yes	[117,118]
	*Iridoviridae*	*Iridovirus*	GbIV	dsDNA	N	B	adult	yes	[57,138]
	*Iridoviridae*	*Iridovirus*	CrIV	dsDNA	N, E	B	adult/nymph	yes	[58,116]
	*Iridoviridae*	*Iridovirus*	Liz_CrIV	dsDNA	E	B	nymph	yes	[60]
	*Iridoviridae*	*Iridovirus*	Cham_IIV	dsDNA	E	B	nymph	yes	[45]
	*Iridoviridae*	*Iridovirus*	IIV-6	dsDNA	N, E	B	nymph	yes	[58]
	*Nudiviridae*	*Alphanudivirus*	GbNV	dsDNA	N, E	B	adult/nymph	yes	[140,141]
	*Parvoviridae*	*Densovirus*	AdDNV	ssDNA	N, E	resistant			[44,90]
*Locusta migratoria*	*Baculoviridae*	*Alphabaculovirus*	SINV	dsDNA	E	B	nymph	yes	[148,149]
	*Iridoviridae*	*iridovirus*	CrIV	dsDNA	E	B	adult/nymph	yes	[116]
	*Iridoviridae*	*iridovirus*	IIV-6	dsDNA	E	B	nymph	yes	[58]
	*Poxviridae*	*Betaentomopoxvirus*	MsEPV	dsDNA	E	B	nymph	yes	[145,146]
	*Poxviridae*	*Betaentomopoxvirus*		dsDNA	N	B	nd	yes	[144]
	*Reoviridae*	*Cypovirus*	CPV	dsDNA	E	B	adult/nymph		[150]
*Schistocerca americana*	*Picornaviridae*	nd	CAV	ssRNA	N, E	B	nymph	yes	[156,157]
	*Poxviridae*	*Betaentomopoxvirus*	MsEPV	dsDNA	N	B	nymph	yes	[154,155]
*Schistocerca gregaria*	*Iridoviridae*	*Iridovirus*	IIV-6	dsDNA	E	B	nymph	yes	[58]
	*Iridoviridae*	*Iridovirus*	CrIV	dsDNA	E	B	nymph	yes	[58]
	nd	nd	Schistocerca virus	nd	N	nd	adult	nd	[151]
	*Poxviridae*	*Betaentomopoxvirus*		dsDNA	N	nd	nd	nd	[152]

Legend: *Autographa californica* multiple nucleopolyhedrovirus (AcMNPV) and two recombinant strains (AcMNPV.AaIT and AcJHE.SG AcMNPV); *Solenopsis invicta* virus 3 (SINV-3); bait-cricket virus (BCV); cricket paralysis virus (CrPV); *Acheta domesticus* iflavirus (AdIV); cricket iridovirus (CrIV); invertebrate iridescent viruses 6 (IIV6); *Acheta domesticus* volvovirus (AdVVV); *Acheta domesticus* mini ambidensovirus (AdMADV); *Acheta domesticus* densovirus (AdDNV); *Penaeus merguiensis* densovirus (PmergDNV); *Gryllus bimaculatus* iridescent virus (GbIV); lizard–cricket iridovirus (Liz_Cr IV); chameleon iridovirus (Cham_IIV); *Gryllus bimaculatus* nudivirus (GbNV); *Spodoptera littoralis* nucleopolyhedrovirus (SINV); *Melanoplus sanguinipes* entomopoxvirus (MsEPV); cytoplasmic polyhedrovirus (CPV); crystalline-array virus (CAV); single strain DNA (ssDNA); double strain DNA (dsDNA); single strain RNA (ssRNA); double strain RNA (dsRNA); natural infection (N); experimental infection (E); bioassay animal (Bi); mechanical vector (M); biological vector (B); not determined (nd).

**Table 4:** Viruses detected in dipteran edible species.

Dipteran Species	Virus Family	Virus Genus	Virus Species	Virus Characteristics	Type of Infection	Vector Status	Stage Involved	Symptoms or Mortality	References
*Hermetia illucens*	none								
*Musca domestica*	*Arteriviridae*	*Betaarterivirus*	PRRSV	ssRNA	N/E	M	adult	no	[224,225,226,227,228]
	*Asfarviridae*	*Asfivirus*	ASFV	dsDNA	N	M	adult	no	[231,232]
	*Circoviridae*	*Circovirus*	PCV2	ssDNA	N/E	M	adult	no	[229]
	*Coronaviridae*	*Alphacoronavirus*	PEDV	ssRNA	E	M	adult	no	[230]
	*Coronaviridae*	*Gammacoronavirus*	TCV	ssRNA	E	M	adult	no	[213]
	*Filoviridae*	*Ebolavirus*	EBOV	ssRNA	E	M	adult	no	[198]
	*Herpesviridae*	*Varicellovirus*	PRV-1	ssDNA	E	M	adult	no	[233]
	*Hytrosaviridae*	*Muscavirus*	MdSGHV	dsDNA	N/E	B	adult	yes	[199,210,240]
	*Reoviridae*	nd	Idno-3	dsRNA	N/E	B	adult/larva	yes	[188,211]
	*Orthomyxovirdae*	*Alphainfluenzavirus*	HPAIV H5N1; LPAI H9N2	ssRNA	N/E	M	adult	no	[212,213,214,215,216]
	*Paramyxoviridae*	*Avulavirus*	NDV; ENDV	ssRNA	N/E	M	adult/larva	no	[218,219,220,221,222]
	*Parvoviridae*	*Amdoparvovirus*	AMDV	ssDNA	N	M	adult	no	[237]
	*Parvoviridae*	*Protoparvovirus*	MEV	ssDNA	E	M	adult	no	[236]
	*Phenuiviridae*	*Phlebovirus*	RVFV	dsRNA	E	M	adult	no	[235]
	*Picornaviridae*	*Enterovirus*	Ento C	ssRNA	E	M	adult/larva	yes	[191,192,193,194,195]
	*Picornaviridae*	*Enterovirus*	Coxs B	ssRNA	N	M	adult	no	[196]
	*Picornaviridae*	*Enterovirus*	Coxs C	ssRNA	E	M	adult	no	[195]
	*Picornaviridae*	*Senecavirus*	SVA	ssRNA	N	M	adult	no	[234]
	*Poxviridae*	*Capripoxvirus*	LSDV	dsDNA	N	M	adult	no	[238]
	*Reoviridae*	*Rotavirus*	SA11	dsRNA	E	M	adult	no	[197]
	*Retroviridae*	*Gammaretrovirus*	REV	ssRNA	N/E	M	adult	no	[223]

Legend: porcine reproductive and respiratory syndrome virus (PRRSV); African swine fever virus (ASFV); porcine circovirus 2b (PCV2); porcine epidemic diarrhea virus (PEDV); turkey coronavirus (TCV); Ebola virus (EBOV); Aujeszky’s virus (PRV-1); *Musca domestica* salivary gland hypertrophy virus (MdSGHV); idnoreovirus 3 (Idno-3); avian influenza virus (HPAIV H5N1; LPAI H9N2); Newcastle disease virus (NDV) and exotic Newcastle disease virus (ENDV); Aleutian mink disease virus (AMDV), Mink enteritis virus (MEV); Rift Valley fever virus (RVFV); poliomyelitis virus (Enterovirus C, Ento C); coxsackievirus B (Coxs B); coxsackievirus C (Coxs C); senecavirus A (SVA); lumpy skin disease virus (LSDV); Simian rotavirus 11 (SA11); reticuloendotheliosis virus (REV); single strain DNA (ssDNA); double strain DNA (dsDNA); single strain RNA (ssRNA); double strain RNA (dsRNA); natural infection (N); experimental infection (E); mechanical vector (M); biological vector (B).

**Table 5:** Viruses detected in lepidopteran edible species.

Lepidopteran Species	Virus Family	Virus Genus	Virus Species	Virus Characteristics	Type of Infection	Vector Status	Stage Involved	Symptoms or Mortality	References
*Achroia grisella*	None								
*Galleria mellonella*	*Baculoviridae*	*Alphabaculovirus*	NPVs	dsDNA	E	B	larva	yes	[261,262]
	*Baculoviridae*	*Alphabaculovirus*	BmNPV	dsDNA	E	B	larva	yes	[263]
	*Baculoviridae*	*Alphabaculovirus*	GmMNPV	dsDNA	N, E	B	larva	yes	[265,266,267,268]
	*Baculoviridae*	*Alphabaculovirus*	HNPV	dsDNA	E	B	larva	yes	[260]
	*Baculoviridae*	*Alphabaculovirus*	ManeNPV-T3	dsDNA	E	B	larva	yes	[264]
	*Baculoviridae*	*Alphabaculovirus*	AcMNPV CIV-MMPs	dsDNA	E	B	larva	yes	[295]
	*Dicistroviridae*	*Aparavirus*	IAPV	ssRNA	N	nd	larva	nd	[288]
	*Dicistroviridae*	*Triatovirus*	BQCV	ssRNA	N	nd	larva	nd	[288]
	*Dicistroviridae*	*Cripavirus*	CrPV	ssRNA	E	B	larva	yes	[286,287]
	*Herpesviridae*	*Simplexvirus*	BHSV-1	dsDNA	E	Bi	larva	no	[254]
	*Iridoviridae*	*Iridovirus*	IIV-6	dsDNA	E	B	larva	yes	[61,270,271,274,275]
	*Iridoviridae*	*Iridovirus*	AgIIV	dsDNA	E	B	larva	yes	[270]
	*Iridoviridae*	*Iridovirus*	IIVs	dsDNA	E	B	larva	yes	[50]
	*Iridoviridae*	*Iridovirus*	SIV	dsDNA	E	B	larva	yes	[283,284,285]
	*Iridoviridae*	*Iridovirus*	TIV	dsDNA	E	B	larva	yes	[269,276,277,278,279,280,281]
	*Iridoviridae*	*Iridovirus*	CrIV	dsDNA	E	B	larva	yes	[61,116]
	*Iridoviridae*	*Iridovirus*	AIV	dsDNA	E	resistant	larva		[282]
	*Iridoviridae*	*Iridovirus*	MIV	dsDNA	E	B	larva	yes	[272,274]
	*Iridoviridae*	*Iridovirus*	IIV-29	dsDNA	E	B	larva	no	[88]
	*Iridoviridae*	*Iridovirus*	IIV-6 recombinant	dsDNA	E	B	larva	yes	[275]
	nd	nd	GFV	dsDNA	N	nd	larva	nd	[290]
	nd	nd	HaV	small RNA	E	B	larva	yes	[291,294]
	nd	nd	Mycovirus A78	nd	N, E	nd	larva	yes	[289]
	*Nodaviridae*	*Alphanodavirus*	NoV	ssRNA	E		larva	yes	[255,256,257]
	*Nodaviridae*	*Alphanodavirus*	PaV	ssRNA	E	Bi	larva	yes	[292,293]
	*Parvoviridae*	*Densovirus*	AdDNV	ssDNA	E	resistant	larva	no	[90]
	*Parvoviridae*	*Densovirus*	GmDNV	ssDNA	E	B	larva	yes	[258]
	*Picornaviridae*	nd	GmclV	RNA	E	B	larva	yes	[51]

Legend: nucleopolyhedrovirus (NPVs); *Bombyx mori* nucleopolyhedrovirus (BmNPV); *Galleria mellonella* nucleopolyhedrovirus (GmMNPV); *Heliothis virescens* nucleopolyhedrovirus (HNPV); *Malacosoma franconicum* nucleopolyhedrovirus (ManeNPV-T3); *Autographa californica* multiple nuclepolyhedrovirus containing invertebrate iridescent virus metalloproteinases (AcMNPV CIV-MMPs); Israeli acute paralysis virus (IAPV); black queen cell virus (BQCV); cricket paralysis virus (CrPV); bovine herpes simplex virus-1 (BHSV-1); invertebrate iridescent virus type 6 (IIV-6); *Anticarsia gemmatalis* invertebrate iridovirus (AgIIV); invertebrate iridescent viruses (IIVs); *Seriscethis* iridescent virus (SIV); tipula iridescent virus (TIV); cricket iridovirus (CrIV); *Apis ceranea* iridescent virus (AIV); mosquito iridescent virus (MIV); *Tenebrio molitor* iridescent virus 29 (IIV-29); invertebrate iridescent virus expressing an insect specific neurotoxin (IIV-6 recombinant); Galleria free virus (GFV); *Heteronychus arator* virus (HaV); Nodamura virus (NoV); Pariacoto virus (PaV); *Acheta domesticus* densovirus (AdDNV); *Galleria mellonella* densovirus (GmDNV); *Galleria mellonella* cell line virus (GmclV); single strain DNA (ssDNA); double strain DNA (dsDNA); single strain RNA (ssRNA); double strain RNA (dsRNA); natural infection (N); experimental infection (E); bioassay animal (Bi); mechanical vector (M); biological vector (B); not determined (nd).

## Data Availability

The data presented in this study are available within this article.

## Supplementary Materials

The following are available online at https://www.mdpi.com/article/10.3390/v13112280/s1, Table S1: Elected articles list: bibliographical details (authors, title, source, publication year, and DOI or PMID) and edible insect species investigated.
